# Gated Bow-Tie Diode for Microwave to Sub-Terahertz Detection

**DOI:** 10.3390/s20030829

**Published:** 2020-02-04

**Authors:** Steponas Ašmontas, Maksimas Anbinderis, Aurimas Čerškus, Jonas Gradauskas, Algirdas Sužiedėlis, Aldis Šilėnas, Edmundas Širmulis, Vladimir Umansky

**Affiliations:** 1 Center for Physical Sciences and Technology, Savanorių ave. 231, 02300 Vilnius, Lithuania; maksimas.anbinderis@ftmc.lt (M.A.); aurimas.cerskus@ftmc.lt (A.Č.); jonas.gradauskas@ftmc.lt (J.G.); algirdas.suziedelis@ftmc.lt (A.S.); aldis.silenas@ftmc.lt (A.Š.); edmundas.sirmulis@ftmc.lt (E.Š.); 2 Vilnius Gediminas Technical University, Saulėtekio ave. 11, 10223 Vilnius, Lithuania; 3 Braun Center for Submicron Research, Weizmann Institute of Science, Rehovot 76100, Israel; vladimir.umansky@weizmann.ac.il

**Keywords:** voltage sensitivity, bow-tie diode, thermoelectric electromotive force, hot carriers, selectively doped semiconductor structure, field-effect transistor, microwave, terahertz frequency

## Abstract

We propose a new design microwave radiation sensor based on a selectively doped semiconductor structure of asymmetrical shape (so-called bow-tie diode). The novelty of the design comes down to the gating of the active layer of the diode above different regions of the two-dimensional electron channel. The gate influences the sensing properties of the bow-tie diode depending on the nature of voltage detected across the ungated one as well as on the location of the gate in regard to the diode contacts. When the gate is located by the wide contact, the voltage sensitivity increases ten times as compared to the case of the ungated diode, and the detected voltage holds the same polarity of the thermoelectric electromotive force of hot electrons in an asymmetrically shaped *n-n^+^* junction. Another remarkable effect of the gate placed by the wide contact is weak dependence of the detected voltage on frequency which makes such a microwave diode to be a proper candidate for the detection of electromagnetic radiation in the microwave and sub-terahertz frequency range. When the gate is situated beside the narrow contact, the two orders of sensitivity magnitude increase are valid in the microwaves but the voltage sensitivity is strongly frequency-dependent for higher frequencies.

## 1. Introduction

Ability of microwave (MW) and terahertz (THz) radiation to penetrate through a low conductivity medium and atmosphere makes it very attractive to be used in many fields, such as high-speed wireless communications [1,2,3], detection of concealed objects [4,5], medicine [6,7], materials science [8,9], and large area imaging [10,11]. Most of the potential microwave and THz applications require sensitive but robust detectors with fast response time operating at room temperature [12]. Field-effect transistors are widely used to sense and detect sub-terahertz and THz radiation [10,13,14,15,16,17,18,19,20]. The possibility to use field-effect transistors as THz detectors was first proposed by Dyakonov and Shur [21]. The detection principle is based on the usage of nonlinear properties of two-dimensional plasma oscillations in a transistor channel. The resulting dc voltage arising between the source and drain is proportional to the power of the incident radiation. It is essential to note that the nonlinear properties of plasma waves in a transistor channel make it possible to detect radiation at frequencies substantially higher than the transistor cut-off frequency since the plasma wave velocity is much higher than the drift velocity of two-dimensional electrons in the transistor channel [21]. A field-effect transistor biased by the gate-to-source voltage and subjected to electromagnetic radiation can produce a dc drain-to-source voltage which has a resonant dependence on radiation frequency ω with a maximum point at plasma oscillation frequency ω_o_ [14,21]. The width of the resonance curve is specified by the inverse of the electron momentum relaxation time, 1/τ_i_. When ω_o_τ_i_ >> 1, the field-effect transistor operates as a resonant detector. On the other hand, when ω_o_τ_i_ << 1, the plasma oscillations are overdamped, and the transistor response is a smooth function of ω representing a non-resonant broadband detection regime. Both resonant and non-resonant regimes of detection have been widely studied [10,13,14,15,16]. It was shown that the resonant detectors are more sensitive than the broadband ones. Currently, the most promising application for imaging and communications appears to be the broadband THz detection at room temperature in the non-resonant regime [22]. Moreover, microwave imaging, as it is known [5], is realized using ultra-wideband radar systems.

Ultra-wideband detection of electromagnetic radiation can be implemented using a bow-tie shaped diode [23,24,25]. Spectral measurements of its voltage sensitivity showed it to be nearly independent of the frequency from 10 GHz up to 0.7 THz [25,26]. The operation principle of the bow-tie diode is based on non-uniform electron heating effects arising due to the specific shape and doping profile of the diode [23]. The bow-tie diode was also used for heterodyne [27] and spectroscopic [28] imaging, for THz radiation detection [29] and sensing [30], and for homodyne spectroscopic THz imaging of concealed low-absorbing objects [31].

It should be noted that all bow-tie diodes have demonstrated lower voltage sensitivity than the field-effect transistors. To increase the sensitivity of the bow-tie diode, we propose a transistor-like design of the bow-tie diode realized as a partial metal cover of either narrow or wide part of the asymmetrically shaped semiconductor structure. In this article, we present an experimental study of detection properties of bow-tie diodes containing metallic gates. The investigation was carried out within the GHz-subTHz frequency range.

## 2. Samples and Measurement Technique

**Figure 1:**
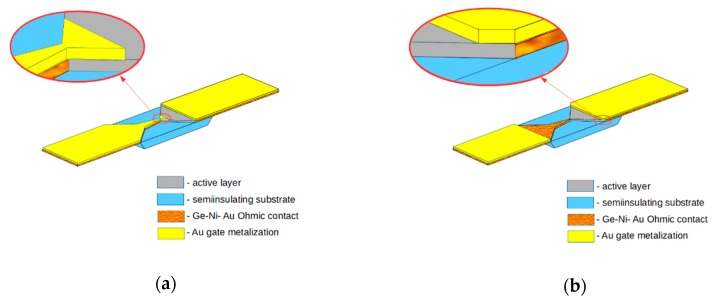
Schematic view of the bow-tie diode with narrow (**a**) and wide (**b**) gate placed over the active layer of the diode. Polyimide film is not depicted in the figure.

The bow-tie diodes with narrow and wide gates above the two-dimensional electron gas channel are schematically depicted in Figure 1.

**Figure 2:**
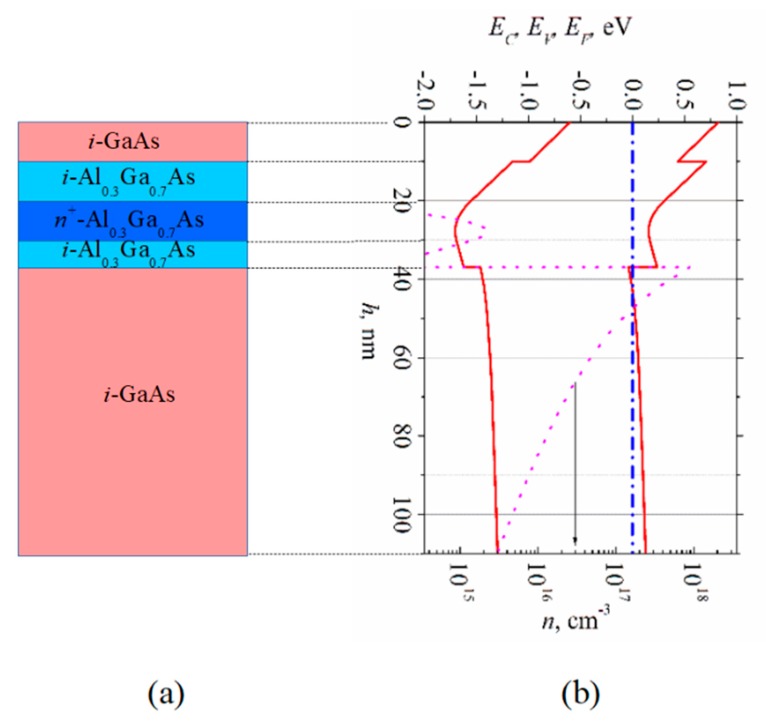
(**a**) Cross-section of selectively doped GaAs/Al_0.3_Ga_0.7_As structure, and (**b**) its energy band diagram with electron density distribution (dotted line).

Selectively doped GaAs/AlGaAs heterostructure epitaxially grown onto semi-insulating GaAs substrate served as the active layer of the diode. The structure was grown in Braun Center for Submicron Research, Weizmann Institute of Science, Israel. A cross-sectional view of the selectively doped structure is shown in Figure 2a. An energy band diagram and electron density distribution in the structure both obtained by means of solving the Poisson equation are depicted in Figure 2b. The sheet of two-dimensional electron gas was formed in *i*-GaAs beside the GaAs/AlGaAs interface. The width of the heterostructure layers (*i-*GaAs cap layer (10 nm), *i*-Al_0.3_Ga_0.7_As spacers (10 and 7 nm), *n^+^*-Al_0.3_Ga_0.7_As doped layer (10 nm)) as well as donor density in the doped layer (3.7 × 10^18^ cm^−3^) were chosen in pursuance of diminishing the parasitic parallel electron gas channel in the AlGaAs barrier and making the selectively doped heterostructure more sensitive to electrical bias voltage applied to the gate which was to be placed on the surface of the structure.

Mesas of the asymmetrically shaped structure were formed using wet chemical etching of the semiconductor in phosphorus acid solution. The etching depth was 35 nm which ensured surefire confinement of the electron channel. The width of the neck in the narrowest part of the diode was 1.5 µm. Ohmic contacts for the diodes were fabricated by thermal evaporation of Ni:Au:Ge:Ni:Au metals of respective thickness 5:200:100:75:100 nanometers onto a photo-resistive mask, and the contact patterns were formed using the lift-off technique. The Ohmic contacts were then finalized by means of rapid annealing in forming gas ambient atmosphere according to the following heating regime: rise to 200 °C/holding 200 °C/rise to 420 °C/holding 420 °C/cooling stage with respective time durations 10 s/60 s/10 s/60 s/150 s. The gates of the diodes were fabricated by applying the lift-off technique for the thermally evaporated Ti:Au metals layer of 15:185 nanometers thickness. Both the wide and the narrow gates spanned the active layer for 8 µm which made up about 20 percent of its length.

**Figure 3:**
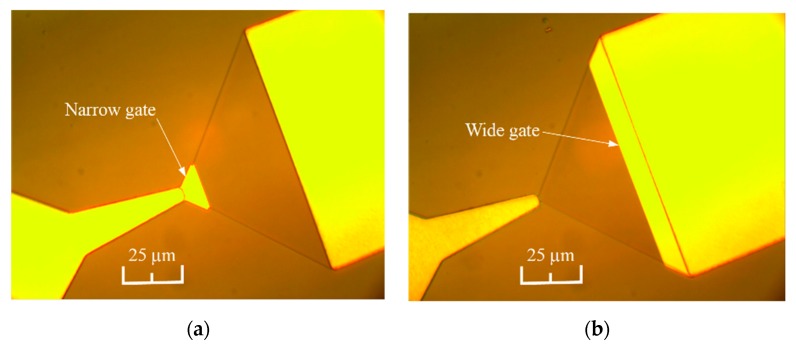
The microphotographs of the bow-tie diodes with narrow gate beside the neck of the diode (**a**) and with the gate near the wide metallic contact (**b**).

The microphotographs of the bow-tie diodes with narrow and wide gates are presented in Figure 3.

Further technological operations were designated to transfer the bow-tie diodes on an elastic dielectric polyimide film. Such a design of microwave diodes is preferable for their use at high frequencies when the dimensions of an MW transmission line drop down to submillimeters. Several micrometer-deep notches were chemically etched around each individual diode. These notches were subsequently used to control the substrate thinning process down to micrometric thickness. The notches also served as aligning marks during the last step of diodes’ fabrication when the metallic contacts had to be stripped off. The face side of the substrate was then covered with polyimide using the spin-on technique and cured at 250 °C temperature in ambient air for one hour. The obtained elastic few-micrometers-thick film served later as mechanical support of the finished diode. Finally, two steps of wet chemical etching were used, first, to thin the substrate from its back side down to several micrometers until the deep notches appeared, and, secondly, to remove the residual semiconductor material from the ends of the metallic contacts.

**Figure 4:**
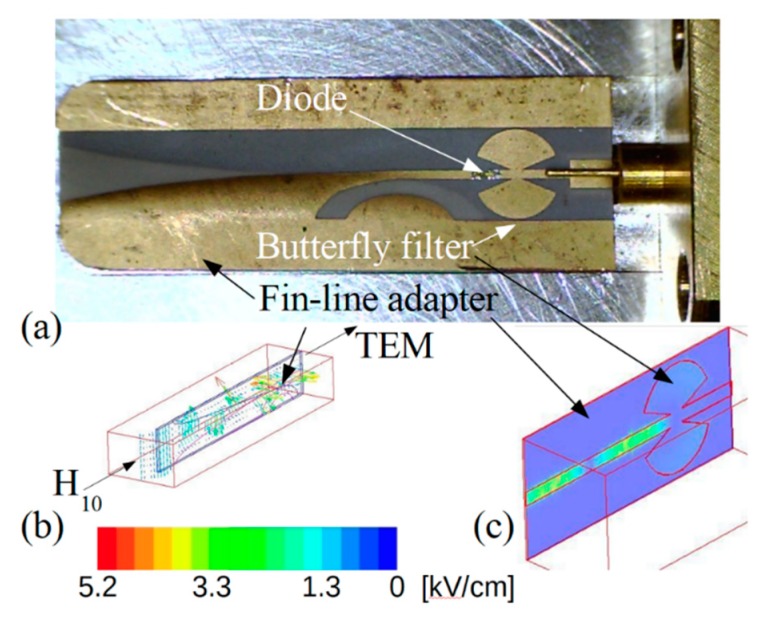
The waveguide head with fin-line adapter (**a**) converting the H_10_ wave propagating in a rectangular waveguide into the TEM wave propagating in the micro strip-line (**b**) containing the butterfly-shaped low-pass filter (**c**).

High-frequency voltage-power characteristics of individual diodes were measured directly using Cascade Microtech high-frequency probe station. Such measurements allowed to investigate electrical parameters of a particular bow-tie diode before and after its gating. High-frequency detection properties of the diodes were investigated by embedding separate microwave diodes into a micro-strip-line (see Figure 4a). A fin-line adapter based on duroid-Cu high-frequency laminate was inserted into the waveguide head to connect the rectangular waveguide with the strip-line. The thickness of the duroid dielectric and Cu metal was 50 μm and 17 μm, respectively. The adapter converted the H_10_ wave propagating in the rectangular waveguide to the TEM wave propagating along the strip-line (see Figure 4b). A butterfly-shaped low-frequency filter (Figure 4c) was used in the fin-line adapter to prevent the microwave signal from coming out of the waveguide head. As sources of microwave radiation, the TWT generators operating in the K_a_ and D frequency bands were used. A signal of microwave radiation at *f* = 300 GHz frequency was generated by taking the second harmonic of the microwave signal at *f* = 150 GHz. The frequency multiplier of Virginia Diodes Inc. was used for this purpose. All the measurements were performed at room temperature.

The investigation of the bow-tie diodes was carried out in the following sequence. First, the voltage-power characteristics of all the ungated diodes still situated on a semiconductor substrate were measured at *f* = 30 GHz using a high-frequency probe station. Later, the gates were formed on the diodes, and spectral measurements were performed on the particular diodes. Finally, after the gated bow-tie diode matrix was transferred onto the polyimide film, the diodes were separated and mounted into the waveguide head, and the frequency dependence of the detected voltage was investigated.

## 3. Results and Discussion

**Figure 5:**
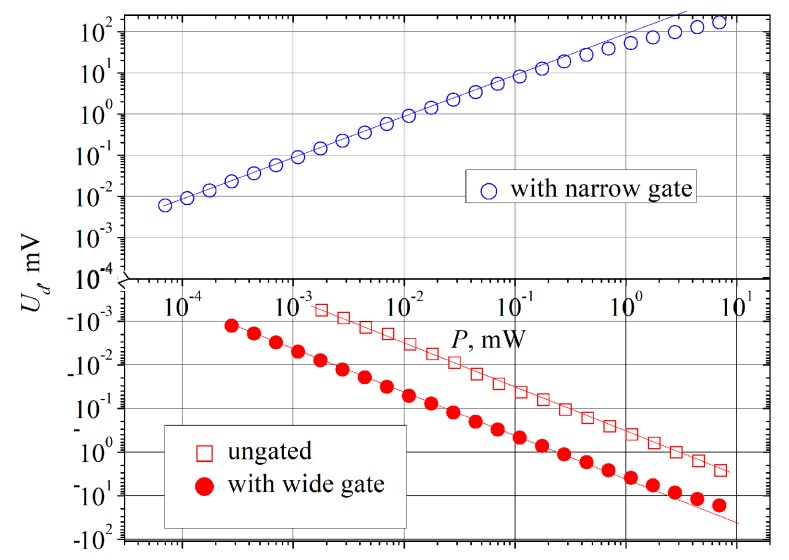
Dependence of the detected voltage on 30 GHz microwave power of the ungated (open red squares) and gated (circles) bow-tie diodes. Open blue circles correspond to the diode with narrow gate, and solid red circles belong to the bow-tie diode with wide gate. The lines are guides for the eye of linear dependence.

Voltage signal detected across the ungated bow-tie diode located on the semiconductor substrate had polarity of the thermoelectric electromotive force of hot electrons, i.e., positive potential was induced on the narrow contact of the diode (left side contact of the diodes depicted in Figure 1). Typical voltage-power characteristics of the diode are presented in Figure 5 by open square dots. Wide dynamic range is one of the desirable features of a detector. As Figure 5 shows, linear dependence of the detected voltage is kept within at least four order-wide microwave power range.

The expression of voltage sensitivity *S_i_*, classically defined as the ratio of the voltage signal *U_d_* detected across the ends of the MW diode and microwave power in a waveguide *P_i_*, was taken from [23,25] and readjusted for the asymmetrically shaped selectively doped semiconductor structure possessing two-dimensional electron gas (2DEG) and containing Ohmic metal–semiconductor junction (the *n^+^-n* junction) in the narrowest part of the structure as:

(1) $$S_{i}=\frac{U_{d}}{P_{i}}=\frac{2R_{sh}\mu_{0}\tan\alpha }{3d^{2}\ln\frac{a}{d}}\frac{P}{P_{i}}N,$$

where *P* is the microwave power absorbed by the diode, *R_sh_* marks the sheet resistance of the diode’s active layer, *μ*_0_ is the low field electron mobility, *a* and *d* indicate the width of the semiconductor structure in the widest and narrowest parts, respectively; *α* is the widening angle of the active layer. The frequency-dependent factor *N* in the case of the weakly and moderately doped semiconductor can be expressed as [25]:

(2) $$\begin{array}{l} N=[\frac{1+{\left(\omega \tau_{M}\right)}^{2}}{{\left(\omega \tau_{M}\right)}^{2}}\{\tau_{E}\left[1+\frac{s^{2}}{1+{\left(\omega \tau_{E}\right)}^{2}}\right]\ln\left[1+{\left(\omega \tau_{M}\right)}^{2}\right]+\tau_{M}\left[\frac{3}{2}-\frac{s(1-s){\left(\omega \tau_{E}\right)}^{2}}{1+{\left(\omega \tau_{E}\right)}^{2}}\right]\times \\ \left[\frac{1}{\omega \tau_{M}}\arctan(\omega \tau_{M})-\frac{1}{1+{\left(\omega \tau_{M}\right)}^{2}}\right]\}+\frac{s(1-s)\tau_{E}}{1+{\left(\omega \tau_{E}\right)}^{2}}]\frac{1}{1+{\left(\omega \tau_{i}\right)}^{2}}. \end{array}$$

**Figure 6:**
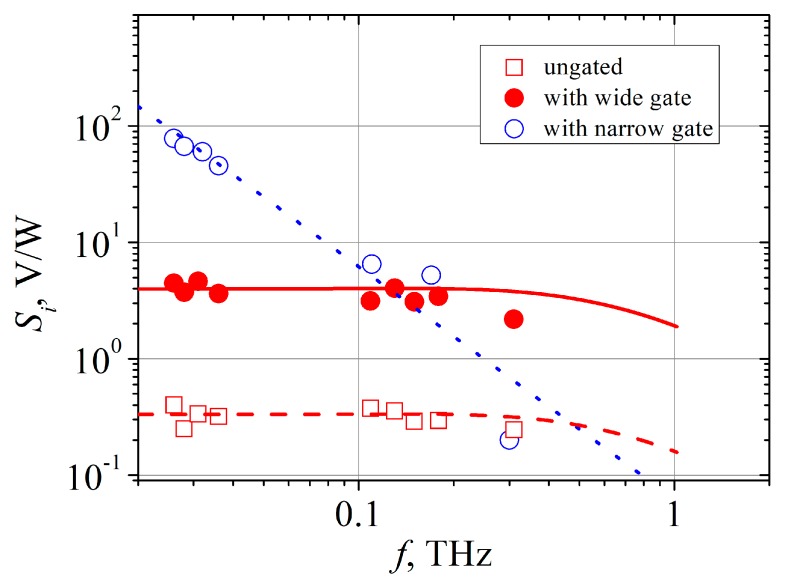
Frequency dependence of the voltage sensitivity of the bow-tie diodes: ungated (open red squares), with wide gate (solid red circles), and with narrow gate (open blue circles). The red dashed line shows dependency of the ungated diode calculated according to Equation (1), and the red solid line calculated according to Equation (5) refers to the wide-gated diode. The blue dotted line is a guide for the eye of 1/ω^2^ dependence.

Here, *s* is the exponent in the dependence of electron momentum relaxation time *τ_i_* on electron energy *E*, *τ_E_* denotes the phenomenological energy relaxation time of an electron, and *τ_M_* stands for the Maxwell relaxation time in the active layer. The measured dependence of voltage sensitivity of the ungated bow-tie diode on radiation frequency is presented in Figure 6, and the calculated dependence of the sensitivity is depicted there by the dashed line. It is seen that the theoretical dependence of *S_i_* on frequency is in good agreement with the experimental data. Noteworthy is the decrease in the voltage sensitivity at higher frequencies. Most probably it is caused by the weakening of electron heating due to microwave radiation as the frequency of the electric field gets higher than the reciprocal momentum relaxation time of electrons. For the calculation, we used electrical parameters of the bow-tie diode which were measured experimentally. The sheet resistance *R_sh_* = 1850 Ω/▯ was measured using the transmission line test structure, and the value of electron mobility at low electric field *μ*_0_ = 5200 cm^2^/(V·s) was obtained by means of Hall measurements. Electron energy relaxation time *τ_E_* = 0.45 ps in n-GaAs at room temperature was taken from [32,33]. Taking into account linear dependence of electron momentum relaxation time on electron energy in GaAs at room temperature, we took the value of the exponent *s* = 1 [25]. The Maxwell relaxation time was calculated using the expression:

(3) $$\tau_{M}=\varepsilon \varepsilon_{0}\rho =\varepsilon \varepsilon_{0}R_{sh}h,$$

where *ε* and *ε*_0_ denote relative permittivity of GaAs and vacuum permittivity, respectively, *ρ* stands for the resistivity of the material, and *h* = 5 nm is the average thickness of the electron gas channel. Electron momentum relaxation time was calculated as:

(4) $$\tau_{i}=\frac{m_{eff}\mu_{0}}{e},$$

where *m_eff_* = 0.0636 *m*_0_ is the electron effective mass in GaAs, and *m*_0_ and *e* note the free electron mass and elementary charge, respectively. For the investigated structure, the electron momentum relaxation time *τ_i_* was 0.19 ps. At lower frequency (in the K_a_ range) the measured ratio *P/P_i_* was equal to 0.02.

After the wide gates were formed onto the same bow-tie diodes, the measurements of voltage-power characteristic and voltage sensitivity of the gated diodes were carried out again at the same frequencies. As Figure 5 shows, the dependence of the detected voltage on microwave power of the wide-gated bow-tie diode is still linear over at least four orders of magnitude of microwave power at 30 GHz (red solid circles). The voltage sensitivity of bow-tie diodes with the wide gate is higher by one order of magnitude than *S_i_* of the ungated diode. Insertion of the wide gate did not change the character of *S_i_* dependence on frequency (see Figure 6). It is known that sensitivity due to THz currents’ rectification by a periodic two-dimensional electron system depends on frequency as [34]:

(5) $$S_{i}(\omega )=S_{i}(0)\frac{1}{1+{\left(\omega \tau_{i}\right)}^{2}},$$

where *S_i_* (0) is the sensitivity at low frequencies. Spectral dependence of voltage sensitivity of the wide-gated bow-tie diode calculated according to Equation (5) and assuming also *τ_i_* = 0.19 ps is depicted in Figure 6 by the red solid line. In the calculation, *S_i_* (0) was taken as 4 V/W to fit the theoretical curve with experimental points at low frequencies. Entering into the terahertz frequency range is marked by a slight decrease of the voltage sensitivity for both wide-gated and ungated bow-tie diodes.

Insertion of the narrow gate essentially changes detection properties of the bow-tie diode. Polarity of the detected voltage becomes opposite as compared to the ungated case (see Figure 5). Measurements of voltage sensitivity’s spectral dependency of the narrow-gated bow-tie diode reveal no presence of plateau, and *S_i_* (ω) decreases according to ω^−2^ law at high frequencies (Figure 6). At low frequencies, its voltage sensitivity exceeds the one of the ungated diode by more than two orders of magnitude, whereas the sensitivities of both diodes become practically equal at 0.3 THz. 

## 4. Conclusions

The impact of gate insertion into the bow-tie diode on its detection properties has been investigated. As for conclusion, the effect of the gate placed above the asymmetrically shaped two-dimensional electron gas channel depends on its location in respect of the diode’s terminals. Experimental results show that the gate does not influence dynamical range of the bow-tie diode. As the gate is inserted by the wide contact, the voltage sensitivity of the detector increases ten times, and it depends on frequency in the same manner as in the case of the ungated diode showing plateau-like constancy up to 0.2 THz. The voltage sensitivity of the diode can be increased by two orders of magnitude at 30 GHz when the gate is formed at the narrow contact. In this case, the polarity of the detected voltage signal changes into the opposite and the frequency dependence of the voltage sensitivity changes its manner and decreases as ω^−2^. As a result, at higher frequencies the preference should be given to the wide-gated bow-tie diode since its voltage sensitivity is much higher.
